# Noninvasive Skin Closure Device (DermaClip®) vs Conventional Sutures: A Randomized Crossover Trial on Cadaver Models

**DOI:** 10.7759/cureus.105321

**Published:** 2026-03-16

**Authors:** Jessica A Martin, Matthew J Perdue, Christopher A Mitchell

**Affiliations:** 1 Department of Emergency Medicine, Carl R. Darnall Army Medical Center, Fort Hood, USA

**Keywords:** combat medic, dermaclip, disease non-battle injury, hollander wound evaluation scale (hwes), laceration, medical simulation training center (mstc), suture, wound closure

## Abstract

Introduction

Lacerations comprise one of the leading causes of disease non-battle injuries encountered both stateside and during deployment. These injuries cause a significant impact on mission accomplishment and readiness, requiring extended time to repair and personnel to manage. The purpose of this study is to compare conventional sutures to the DermaClip^®^ noninvasive skin closure device (DermaClip US, LLC, Houston, TX, USA) on cadaver models. The hypothesis is that there will be a difference in the time of skin closure between DermaClip^®^ and sutures. The primary outcome of the study is to compare the time of skin closure between the two methods, while the secondary outcomes will assess the efficacy and user confidence for each method.

Methods

We conducted this study in the Medical Simulation Training Center (MSTC) cadaver lab at Fort Hood, TX, USA. The study was performed during a biannual cadaver procedure lab on five unembalmed, fresh-frozen cadavers. The UTHealth Houston provided cadavers previously donated by the decedent before death or by the decedent’s family. We recruited volunteers from the Carl R. Darnall Army Medical Center ED residents, staff physicians, and students participating in MSTC training. Volunteers received a block of instruction on the placement of the DermaClip^®^ skin closure device from the manufacturer’s website. Investigators randomly assigned volunteers to two groups and timed skin closure utilizing DermaClip^®^ or conventional sutures. A study team member verified the skin closure and assigned a grade utilizing the 6-point Hollander Wound Evaluation Scale. Volunteers were also asked to complete a survey to rate the confidence and efficacy of each method.

Results

Twenty participants performed 40 laceration repairs using either the DermaClip^®^ or conventional sutures. They conducted 20 repairs using DermaClip^®^ and 20 using conventional sutures. Our results demonstrated a significant time difference in skin closure time, favoring the DermaClip^®^ device over conventional sutures (77 seconds vs 456 seconds, P = 0.001).

Conclusions

This study sought to detect whether a significant difference in time to skin closure exists between DermaClip^®^ and conventional sutures. Secondarily, we sought to evaluate efficacy and confidence. Following analysis, DermaClip^®^ demonstrated significantly less time to skin closure. However, although participants rated DermaClip^®^ higher in efficacy and confidence, the results were not significant. Further studies are required, preferably on live patients with a cost analysis, to determine if DermaClip^®^ is superior to conventional sutures.

## Introduction

Lacerations are a common complaint addressed by ED providers. The time dedicated is costly for institutions, and the associated risk is high due to needlestick injuries estimated to cost $650-$750 per incident [1]. Laceration repair with sutures requires additional anesthetic medication and equipment to complete, which makes it a costly procedure. In the ED, the price ranges from as low as $24 without complications to as high as $69 if the patient develops an infection or wound dehiscence [2]. This also remains true in the military, with costs, including supplies, ranging from $42 to $52 per repair taken from the Defense Medical Logistics Standard Support acquisition website.

In the Armed Forces, lacerations are predominantly classified as disease non-battle injuries (DNBIs). In many conflicts throughout history, DNBIs have often resulted in more lost person-days than combat injuries [3]. Lacerations comprise one of the leading causes of DNBIs encountered while deployed and make up one of the most common chief complaints encountered in theater [3]. They have a significant impact on mission accomplishment and combat readiness and require extended time to repair and personnel to manage. A study evaluating the utilization of the 28th Combat Support Hospital during Operation Inherent Resolve showed that non-battle injuries and illnesses were the predominant reason for ED utilization, with laceration repair being the second most prevalent procedure performed [4]. Therefore, it is essential for both prehospital and hospital providers to be skilled in multiple laceration repair techniques.

In the military prehospital setting, the medic, operating independently or in support of the physician or physician assistant (PA), represents the first medical provider delivering tactical combat casualty care in the contested operational environment [5]. They have various degrees of training, limited supplies in their aid bags, and their wound closure experience varies. Although all medics complete an advanced individual training program, their subsequent clinical proficiency varies considerably. Differences arise from variability in unit assignment, dictating access to sustainment training, procedural exposure, leadership emphasis on skills validation, and opportunities for supervised hands-on experience. For example, some medics assigned to military treatment facilities routinely perform wound irrigation, closure techniques, and advanced airway adjunct placement under supervision, while those assigned to operational units get limited procedural repetition outside of simulation-based training. The unit PA facilitates their training, emphasizing their individual critical task list, focusing on massive hemorrhage, airway, respirations, and shock management, but it does not require competency in suture repair for junior medics.

Logistical constraints further compound this variability. Supplies are limited to what is carried in an aid bag, which is intentionally configured to address the leading preventable causes of death on the battlefield [6]: massive hemorrhage, airway obstruction, impaired respirations, and shock. As a result, medics may lack both the equipment and the validated competency to perform laceration repair at the point of injury. The combined effect of inconsistent procedural exposure and limited resources increases the likelihood of combat medics evacuating simple non-life-threatening lacerations to higher echelons of care, potentially increasing evacuation burden, delaying return to duty, and diverting medical assets from more critically injured casualties.

Laceration repair is completed with multiple different methods, including sutures, adhesive strips, topical skin adhesives (TSAs), staples, and noninvasive devices. The advantages of sutures are their strength and flexibility, which allow the closure of simple and complex lacerations at various anatomical sites. Although sutures offer many benefits, they also have drawbacks. The most significant drawback remains that suture methods consume a considerable amount of time, with the added inconvenience of removal after the healing process [7]. Despite these limitations, sutures remain the intervention of choice for laceration repair.

Presently, the Armed Forces are transitioning to large-scale combat operations (LSCO) and are preparing for larger numbers of casualties than seen in recent conflicts in Iraq and Afghanistan. Current casualty estimates range between 50,000 and 60,000 casualties per 100,000 personnel, which will cause a significant impact on lethality and combat effectiveness [5]. Considering that the projected conflicts will be against near-peer adversaries, this limits the ability to evacuate casualties for prolonged durations. It is crucial to identify a superior cutaneous wound closure device that can be efficiently and effectively used both in the field and in a hospital setting to ensure timely and effective treatment of cutaneous injuries, as well as to expedite surgical closures [8].

The DermaClip^®^ device (DermaClip US, LLC, Houston, TX, USA) should be considered to address the limitations associated with conventional sutures. The manufacturers promise improved application times, no need for local anesthesia, and elimination of follow-up encounters for removal. The ease of application requires minimal training to complete laceration repair, potentially broadening treatment capabilities in austere or resource-limited environments. Since lacerations remain one of the most common procedures performed in EDs across the United States, with over six million treated yearly [2], innovations that improve efficiency are clinically relevant. In the military, lacerations are a common injury seen during both training and deployments. These injuries historically require evacuation to the nearest role of care for definitive management because, currently, the gold standard for laceration repair is sutures, which require significant time to accomplish, additional equipment, and medications. Suture closure takes approximately 10 times longer than staples and four times longer than skin adhesives, not including the time required for local anesthesia [8]. To address this gap, new methods that are effective, rapid, and efficient and allow soldiers to continue the mission must be investigated.

Noninvasive closure methods like DermaClip^®^ potentially address these shortcomings and may offer a practical alternative. However, important limitations must be considered. The device is contraindicated in patients with known adhesive allergies and should not be used on infected wounds. It is also unsuitable for wounds under excessive tension, wounds that cannot be approximated, or anatomical locations where the device cannot fully adhere to intact surrounding skin. Application along the hairline requires prior hair removal to ensure adequate adhesion. These limitations may restrict its use in certain operational or clinical scenarios and should be weighed when determining appropriateness for laceration management.

The military is in the midst of transitioning from counterinsurgency operations to LSCO, where medical personnel will have limited supplies and face longer evacuation times. The roles of medics will expand, and they will be responsible for independently treating or assisting in managing multiple casualties spontaneously while providing prolonged field care, which includes laceration repair. They must be equipped with supplies that allow them to address minor injuries to facilitate returning soldiers to duty in this evolving battlefield environment.

Providers develop suturing proficiency over years of practice, which leads to significant variations in skill levels. All emergency medicine providers develop their skills throughout medical school, residency, and practice, yet variation in expertise persists. In the military, medic suture training is administered by their supervising provider, and many medics only have a few hours of total hands-on suture training. This is why laceration repair by a medic typically requires supervision. In the evolving battlefield, medics need to be able to complete laceration repair properly and without supervision. Noninvasive methods like DermaClip^®^ bridge the gap in experience and empower medics to perform laceration repair independently.

The pursuit of improved laceration repair techniques remains a significant focus in surgery, emergency medicine, and the military. Many studies explore laceration repair options, evaluating complications, cosmesis, patient satisfaction, and comparisons among sutures, staples, skin adhesives, and Zip surgical devices. Current research suggests that TSAs result in a shorter ED length of stay than sutures or staples [9]. However, the authors identified an absence of literature evaluating DermaClip^®^ compared to conventional laceration repair methods. Currently, no studies compare the DermaClip^®^ noninvasive skin closure device to sutures in terms of primary skin closure time, although multiple studies have compared sutures with skin adhesives and Zip surgical devices.

Studies evaluating optimal methods for laceration repair demonstrate variable results, and sutures remain the primary technique for closing wounds [10]. Various incision closure techniques have been applied in clinical practice, including synthetic sutures, absorbable sutures, staples, and adhesive compounds [11]. Two noteworthy articles report mixed reviews, attributing strengths and weaknesses to each option without identifying a superior technique [12,13]. The development of noninvasive methods is not new and has been utilized in medicine over the last decade. Studies on the Zipline surgical closure device report advantages in infection reduction, time savings, and cosmesis [14]. Due to evidence suggesting the superiority of noninvasive devices, DermaClip^®^ was developed to address shortcomings identified with its predecessors [15]. Our study will evaluate DermaClip^®^ by comparing it to sutures for time of skin closure as the primary outcome. Secondary outcomes included participants’ opinions on their confidence in laceration repair skills pre- and post-study and their appraisal of the efficacy and confidence in each device.

In 2020, Xie et al. [14] conducted a meta-analysis comparing efficacy, wound dehiscence, total wound complications, wound closure time, and scar score between sutures and a zipper device following surgery. The authors reported that the zipper device was superior in multiple areas, most significantly decreased surgical site infections (SSIs) and wound closure time. They hypothesized that the therapeutic effects occur due to the noninvasive approach and decreased surgical time, which limits bacterial inoculation opportunities. The authors emphasized the simplicity of the zipper device, eliminating the need for advanced surgical skills, reducing technical variation among surgeons, and promoting standardization of wound closure. They provided examples of practical applications, noting that zipper devices in overcrowded EDs could improve wait times, leading to economic benefits for patients who can remove them without follow-up encounters. The study suggested distinct advantages for African Americans by reducing skin trauma and potentially decreasing keloid formation. It also provided a painless alternative to suture repair for pediatric patients and eliminated the need for local anesthesia. The authors noted that no sharps are required during anesthesia and closure, reducing the risk of needle puncture accidents and associated institutional costs.

The study’s findings are encouraging, suggesting noninvasive wound closure methods are superior to sutures and provide numerous advantages for institutions and diverse patient populations, with added risk-reduction benefits to practitioners. However, the meta-analysis had limitations, including sample size and inclusion of a nonrandomized control trial, introducing potential bias. Additionally, studies included compared different suture types (subcuticular vs non-subcuticular) and materials, limiting generalizability. More extensive and rigorous studies are needed to confirm these findings and assess long-term complications in clinical practice.

The study by Koerber et al. [16] evaluating the Zipline surgical device reported findings consistent with Xie et al. [14], showing reduced closure times and no postoperative complications or infections. Koerber et al. [16] conducted a single-center, retrospective cohort study of 175 patients at the University of Missouri who received cardiac implantable electronic devices (CIED) from October 2015 to April 2017. Primary outcomes were total procedure and pocket closure time, with infection rate as a secondary outcome.

Koerber et al. [16] concluded that the ZIP noninvasive surgical skin closure device significantly shortened pocket closure and procedure times compared to standard sutures without increasing CIED infection rates. The study revealed that closure time for implantable cardioverter-defibrillators was longer than for permanent pacemakers with sutures, but this difference was not observed with the Zip device. They also noted that incision length does not increase application time with the Zip device, allowing extensive wounds to be closed efficiently. Findings support its use in busy EDs to improve procedure time and overall length of stay. Although decreased surgical site and pocket infections were observed, the differences were not statistically significant due to the small sample size. Nonetheless, the findings support the hypothesis from Xie et al. [14] that noninvasive techniques limit bacterial entry and preserve dermal integrity through equal pressure distribution and adhesive application. Limitations include a small sample size and a focus on CEID procedures, limiting generalizability to broader patient populations or traumatic injuries.

Lewis et al. [17] conducted a prospective randomized trial comparing TSA and nylon sutures (NSs) for forefoot incision closure. The primary outcome was wound cosmesis assessed two weeks postoperatively using the Hollander Wound Evaluation Scale (HWES) [18]. Secondary outcomes included time for wound closure, assessment, cosmesis satisfaction, wound pain, and infection rate. TSA and NSs both provided high satisfaction rates, low pain scores, and low complications [17]. However, TSA showed more wound inflammation and dehiscence, associated with increased cost and operative time. The authors recommended that while TSA is a reasonable alternative, it is inferior to interrupted sutures in forefoot surgery. They also hypothesized that TSA’s limitations were related to adhesive differences compared with Zip devices.

Although studies involving the noninvasive Zip device are encouraging, minimal data currently evaluate the DermaClip^®^ device. As laceration repair continues to be time-intensive in crowded EDs, medical device manufacturers will continue developing alternatives to streamline the process. Future studies are needed to assess clinical benefit, patient outcomes, cost-effectiveness, and institutional advantages with the adoption of noninvasive techniques like DermaClip^®^. Of the three key studies reviewed, two compared sutures with a noninvasive skin closure device, while the third compared skin closure with sutures and skin adhesives on an extremity. Two additional studies evaluated DermaClip^®^: one was a case report on performance in fragile skin, and the other compared it to current wound closure techniques; these were excluded from the literature review because the authors are members of the company’s medical advisory board.

## Materials and methods

**Figure 1:**
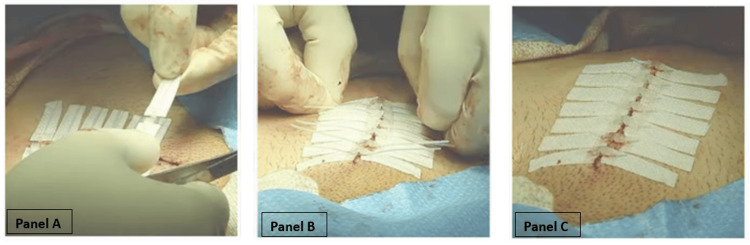
Wound closure process with DermaClip® Panel A: DermaClip^®^ being applied to the skin. Panel B: Closing the wound following DermaClip^®^ application. Panel C: Completed wound closure with DermaClip^®^ in place. Images sourced from www.dermaclip.com with permission from DermaClip US, LLC (Houston, TX, USA)

**Figure 2:**
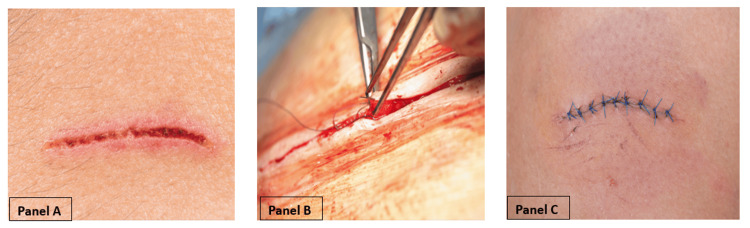
Wound closure with sutures Panel A: Open laceration requiring suture repair. Panel B: Wound closure being completed with sutures. Panel C: Completed wound closure using the suture method. Images purchased from Adobe Stock: Panel A (Adobe Stock #84938503), Panel B (Adobe Stock #215021282), and Panel C (Adobe Stock #165376463)

This prospective, randomized crossover study was approved by the Carl R. Darnall Army Medical Center (CRDAMC) Human Research Protections Office (HRPO) as an institutional review board-exempt study (CRDAMC.23-51/eIRB reference # 963969). The study evaluated the time of laceration repair using DermaClip^®^ (noninvasive skin closure device) (Figure 1) and conventional sutures (Figure 2).

The study design was chosen for resource efficiency and to allow participants to serve as their own control. This is important because we are comparing medical personnel with a wide variety of experience, training, and skills to medics. The crossover design removes experience and ability from the equation by comparing participants to themselves. The authors hypothesized that there would be a difference in time to wound closure when comparing DermaClip^®^ and sutures.

Data collection occurred during a designated cadaver lab at the Fort Hood Medical Simulation Training Center (MSTC). Participants performed each intervention while being timed by an investigator, and results were recorded. The study’s primary outcome was the time to skin closure using either DermaClip^®^ or sutures. A research team member recorded the time and evaluated the skin closure using the HWES [18] after the participant reported completion. Secondary outcomes included participants’ opinions on their confidence in laceration repair skills and their appraisal of the efficacy and confidence in each device.

The primary independent variable was the device used for skin closure (DermaClip^®^ or sutures). The primary dependent variables included closure time, efficacy, and confidence. Each participant had a 10-minute time limit with each device to achieve skin closure. The time limit started when the research team member verbalized “go” and ended when the participant verbalized completion. The 10-minute limit was derived from Lewis et al. [17], which compared TSA vs NSs and reported closure times ranging from 6.8 to 11.1 minutes; 10 minutes was chosen as a practical midpoint.

This study compared DermaClip^®^ vs conventional sutures on cadaver models. A convenience sample of active-duty Army medical personnel volunteers participating in the cadaver lab at Fort Hood, TX, USA was enrolled. No prior experience with DermaClip^®^ was required, but participants were required to have baseline knowledge of performing laceration repair. The study demographic represented an active-duty military population, excluding medical professionals younger than 18 or older than 65 and those with physical limitations preventing laceration repair. The majority of volunteers (13) were combat medics attending MSTC cadaver training, with 10 having 1-4 years of suturing experience (50% of the study population), 30% with no experience, and the remaining 20% comprising Army medical providers with 5-20 years of suturing experience. No personnel from other service branches (Navy, Air Force, etc.) were included, as no other branches were present during data collection at this Army installation.

Twenty participants were enrolled and randomly assigned to start with either DermaClip^®^ or sutures. Device order was determined using an online randomization generator (www.random.org). The study population included Army combat medics (68W), certified PAs, and emergency medicine physician residents and staff. Five cadavers were used for the study; no demographic information was recorded for the cadaver models. All study activities were performed at MSTC during the biannual cadaver procedure lab.

Before starting, participants received a briefing on study expectations, procedures, and timeline. Investigators demonstrated how to apply DermaClip^®^ according to manufacturer recommendations. A 15-minute practice period was provided for all participants to familiarize themselves with both methods. Investigators pre-marked 6 cm vertical lacerations on the upper and lower extremities using a ruler before data collection. The initial method of closure for each participant was randomly assigned to reduce recall bias. All materials were pre-set at the bedside prior to each iteration.

The time to complete the skin closure started with the command “go,” and the participant performed closure with their assigned method. Upon completion, participants verbalized “complete,” and investigators stopped the timer, recording minutes and seconds. An investigator then assessed closure adequacy using the HWES [18]. The cadaver station was reset, and closure was then completed with the other method, following the same procedure. Participants were allowed a maximum of 10 minutes for each repair; the study iteration was terminated if this limit was exceeded. Upon completing both iterations, participants completed a post-participation survey assessing confidence in performing laceration repair and evaluating the efficacy and confidence for each device. No follow-up was required.

The principal investigator (PI) maintained all data in opaque envelopes until transferring it to a Microsoft Excel spreadsheet (Microsoft Corporation, Redmond, WA, USA) on a government computer. Data were stored securely behind a locked drawer when not in use. No PII or PHI was collected; demographic information included only gender, professional status, suturing experience, and years of practice.

A pre-study power analysis was conducted in collaboration with Baylor University statisticians. The study by Koerber et al. [16] was used for effect size estimates due to the lack of prior studies comparing DermaClip^®^ to conventional sutures. It evaluated CIED pocket closure times and infection rates between the noninvasive Zip Surgical Skin Closure device (ZIP) and subcuticular sutures, finding a mean closure time of 14.9 ± 6.8 minutes with the ZIP device vs 20.1 ± 11.09 minutes with sutures. We calculated paired data effect sizes, with alpha = 0.05, beta = 0.8, and a 95% CI. A difference of 5-5.5 minutes was considered clinically meaningful. Assuming normality and using a paired t-test, the number of pairs required ranged from 9 to 16, for a total sample size of 18-32.

All collected data and demographic information were entered into Microsoft Excel on a government-protected laptop. Statistical analyses were performed using R version 4.1.1 [19]. Fisher’s exact tests were used to evaluate whether the order of performing the intervention affected responses; all P-values were >0.05, indicating no association. Primary outcomes were summarized using means and SDs. Closure times were analyzed using the Wilcoxon rank-sum test due to non-normal distribution. Secondary outcomes, measured on a 5-point Likert scale, were analyzed using the paired Wilcoxon signed-rank test, without assuming normality.

The hypothesis was accepted if data analysis demonstrated ≥95% accuracy, defined as the percentage of true positive and negative interpretations.

## Results

This study sought to evaluate the time required for laceration repair using DermaClip^®^ vs sutures. We also assessed each user’s confidence in the devices, their ability to perform repairs, and the perceived efficacy of each method. Recruitment, enrollment, and data collection occurred from August 22 to 25, 2023, with 20 participants: 13 Army medics, one PA, one resident, two staff physicians, and three other medical professionals.

On each day of data collection, the PI provided a brief presentation, including a demonstration for the participants, obtained informed consent, and reviewed study requirements. Each participant completed two laceration repairs, one using each method, for a total of 40 repairs. Participants were randomized into one of two groups using an online randomization generator (www.random.org): participants assigned an even number began with DermaClip^®^, while those assigned an odd number began with sutures. Participant demographics, time measurements, and questionnaire responses were recorded in a Microsoft Excel spreadsheet. All participants completed the study, and none requested to withdraw.

During data collection, 20 medical personnel used both interventions to perform laceration repair. The majority of participants were Army personnel (primarily aged 18-34), with ranks E4-E6, and Military Occupational Specialty 68W. Demographics are summarized in Table 1, both for the overall sample and by intervention order. Key measured outcomes included participants’ self-assessed confidence in using each method and their appraisal of each method’s efficacy.

**Table 1:** Summary of demographics n = total number of participants evaluated in each category; reflects the overall number broken down by the order in which they completed closure using each method and by demographic category; S-D = order of closure completed was suture first, then DermaClip^®^; D-S = order of closure completed was DermaClip^®^ first, then suture; P-value = results of statistical analysis performed using Fisher’s exact test

Crossover order	Overall, N = 20	S-D, N = 10	D-S, N = 10	P-value
Age				0.3
18-24	9 (45%)	6 (60%)	3 (30%)	
25-34	9 (45%)	3 (30%)	6 (60%)	
35-44	1 (5%)	1 (10%)	0 (0%)	
45-54	1 (5%)	0 (0%)	1 (10%)	
55-64	0 (0%)	0 (0%)	0 (0%)	
Gender (male/female)	12 (60%)/8 (40%)	6 (60%)/4 (40%)	6 (60%)/4 (40%)	>0.9
Rank				0.2
E1-E3	2 (10%)	2 (20%)	0 (0%)	
E4-E6	13 (65%)	7 (70%)	6 (60%)	
E7-E9	0 (0%)	0 (0%)	0 (0%)	
O1-O3	3 (15%)	0 (0%)	3 (30%)	
O4-O6	2 (10%)	1 (10%)	1 (10%)	
Suture experience (years)				>0.9
None	6 (30%)	3 (30%)	3 (30%)	
1-4 years	10 (50%)	5 (50%)	5 (50%)	
5-8 years	1 (5%)	1 (10%)	0 (0%)	
9-12 years	1 (5%)	1 (10%)	0 (0%)	
13-16 years	1 (5%)	0 (0%)	1 (10%)	
17-20 years	1 (5%)	0 (0%)	1 (10%)	

There were nearly equal numbers of males and females, with most subjects having minimal suture experience (80% with none or 1-4 years). The Fisher’s exact test was used to determine whether there was an association between the order of interventions and demographics, consistently yielding P-values > 0.05, supporting the null hypothesis. Although the sample size is small, there is no evidence of differences in these variables between the two intervention orders.

We examined time to skin closure as our primary outcome of interest. Analysis revealed that the time distributions were far from normal due to truncation at 0 and 10 minutes. To account for this, we applied a nonparametric test. Summary statistics and test results are presented in Table 2. As hypothesized, time to closure was significantly faster using DermaClip^®^ compared with sutures (P < 0.001).

**Table 2:** Summary of outcomes by method Mean (SD); n (%); Mean (SD) = shown in parentheses as the SD; represents closure time overall and broken down by method (sutures vs DermaClip^®^) when evaluating time to skin closure; n (%) = the percentage of participants receiving the corresponding HWES [18] score, reported overall and by method (sutures vs DermaClip^®^); P-value = represents the statistical test used: Fisher’s exact test for time to skin closure and Wilcoxon rank sum test for HWES [18] results. HWES, Hollander Wound Evaluation Scale

Intervention method	Overall, N = 40	Suture, N = 20	DermaClip, N = 20	P-value
Time of skin closure	4.27 (3.72)	7.36 (2.78)	1.17 (0.73)	<0.001
HWES score				0.3
0	20 (50%)	7 (35%)	13 (65%)	
1	5 (13%)	4 (20%)	1 (5%)	
2	5 (13%)	3 (15%)	2 (10%)	
3	2 (5%)	1 (5%)	1 (5%)	
4	7 (18%)	5 (25%)	2 (10%)	
5	1 (2.5%)	0 (0%)	1 (5%)	

**Figure 3:**
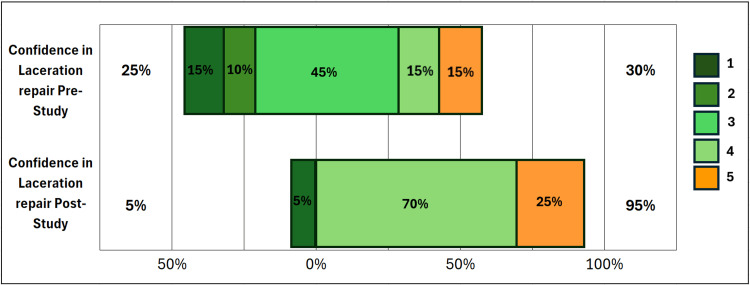
Confidence in skills before and after study Confidence in laceration repair pre-study = the graph shows that the majority of participants were not comfortable with their skills prior to the study (70%). Confidence in laceration repair post-study = the graph shows a shift in overall confidence, with 95% of participants reporting improved confidence in their repair skills following study completion.

**Figure 4:**
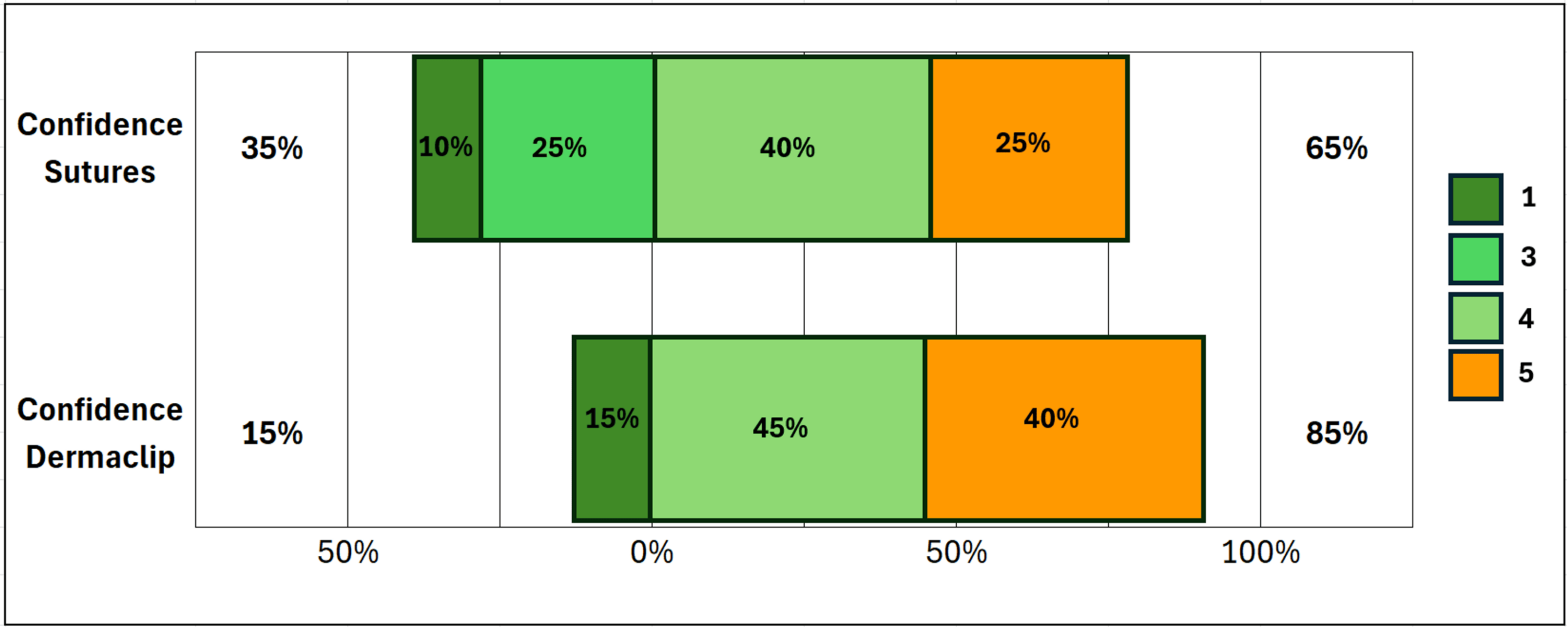
Confidence in methods (sutures vs DermaClip®)

**Figure 5:**
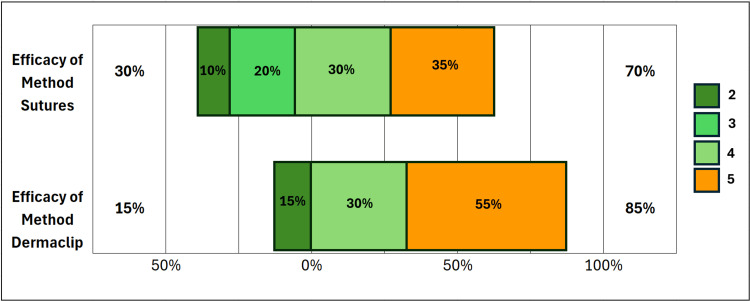
Efficacy ratings for methods (sutures vs DermaClip®)

The additional measured outcomes in the study include participants’ ratings of the efficacy and confidence for the two methods. These efficacy and confidence variables were rated on a Likert scale, with 1 = not at all confident (very dissatisfied with efficacy) and 5 = very confident (very satisfied with efficacy), as reflected in Figure 3, Figure 4, and Figure 5.

We observed a significant increase in confidence in laceration repair skills post-participation compared to pre-participation, 95% vs 30%, respectively (P < 0.00046) (Figure 3). Similarly, we compared confidence between the two methods (Figure 4). Although DermaClip^®^ demonstrated increased confidence, this did not reach statistical significance using the Wilcoxon Signed Rank test (P = 0.13). We observed similar results for efficacy ratings between the two methods (Figure 5), with DermaClip^®^ slightly higher but not statistically significant (Wilcoxon signed rank test, P = 0.1767).

Given the crossover design of our study and the potential risk of a carryover effect, we evaluated this possible confounder using an ANOVA model. The outcome (response) was closure time. The model included Method (DermaClip^®^ or sutures, our primary interest), Order (S-D or D-S), and Period (intervention 1 or intervention 2). Order was not significant (P = 0.876), suggesting no carryover effect. Similarly, Period was not significant (P = 0.824), indicating that intervention timing was also not a factor. Controlling for these potential effects, the method remained highly significant (P < 0.0001). We can therefore conclude that the difference in closure time is truly significant.

## Discussion

This study aimed to compare DermaClip^®^ to conventional sutures in terms of time to complete laceration repair and to evaluate medical personnel’s opinions on confidence pre-/post-study and efficacy. Twenty participants completed 40 laceration repairs. Results show that DermaClip^®^ significantly reduces closure times when compared to sutures. The time to completion for DermaClip^®^ averaged 77 seconds, and conventional sutures averaged 456 seconds, reducing the time by an average of 6.18 minutes. There was also improved confidence in laceration repair skills overall post-study, but no difference in confidence was found for each of the evaluated methods. Participants rated DermaClip^®^ slightly higher in efficacy, but the differences were not significant. We also did not identify any carryover effects that influenced the results when analyzing using the ANOVA method.

This study details the time differences in laceration repair and medical personnel's views on confidence/efficacy between DermaClip^®^ and conventional sutures. The progression of wound closure methods, specifically those that are noninvasive, promises substantial advancements in wound repair. This research, alongside studies like that of Xie et al. [14] and Koerber et al. [16], showcases that utilizing noninvasive skin closure methods provides the advantages of anti-infection, time-saving, and cosmesis for wound closure [14].

To our knowledge, this is the first randomized crossover study directly comparing DermaClip^®^ to sutures, measuring the time of laceration repair. During our literature review, we identified studies that assessed the time difference between sutures and a noninvasive novel zipper device and skin adhesives. Although these studies primarily assessed either SSIs or HWES [18], with time being considered a secondary outcome, we focused solely on time as our primary outcome. While there is a lack of publications specifically evaluating DermaClip^®^, we found a military study by Freed and Ko that reviewed the Chinese Clinical Trial of Medical Devices data with unbiased US-based statisticians [15] and one other study evaluating its performance on fragile skin, but both had authors on the DermaClip^®^ medical advisory board. However, the Freed and Ko study did not evaluate time as an outcome, but success in wound healing, clinical efficacy, ease of operation, postoperative care, and scar results were all found not to be significant.

The study by Lewis et al. [17] found that tissue adhesives received a higher HWES [18] at two weeks post-op and had longer skin closure times but had less reported pain, with superior patient cosmetic satisfaction. These findings suggest that tissue adhesives prolong procedure time and do not provide the strength of sutures. The DermaClip^®^ is a combination device that uses hypoallergenic skin adhesive applied to latex strips with plastic closures. The skin adhesives on the device do not require drying time, which influenced the findings in Lewis et al. [17]. Our study found DermaClip^®^ significantly faster than conventional sutures in laceration repair and received a lower HWES [18] on cadaver tissue. Additionally, we found a significant improvement in confidence in laceration repair skills in preparticipation compared to postparticipation, not reflected for a specific method in post-survey data [14].

Xie et al. [9] conducted a systematic review and meta-analysis comparing the efficacy of the zipper device and sutures for wound closure after surgery [9]. The meta-analyses by Xie et al. [9] included seven randomized controlled trials and one nonrandomized control trial, of which only five trials with 639 patients reported skin closure times. Both their studies and ours found a significant difference in the time of skin closure with the noninvasive method. In our study, the majority of participants were combat medics with variable experience in laceration repair, which could account for the extended suture time.

Additionally, our study evaluated user views on efficacy and confidence in laceration repair pre/post-study as secondary outcomes. We found that participants’ evaluation of efficacy and confidence was higher for DermaClip^®^ vs conventional sutures, but not statistically significant.

The findings of these two studies suggest that noninvasive skin closure methods lead to better patient satisfaction, decreased postoperative pain, and shorter procedure times. Our study highlights the decreased time for repair with minimal experience, which makes an argument for further evaluation in austere environments, EDs, and troop medical treatment facilities.

Limitations of the study

The main objective of this study is to compare two methods of laceration repair by analyzing data on completion time and participants’ thoughts on the efficacy and confidence before and after completion. The investigation team identified several limitations to this study. The authors believe that some limitations may have caused the lack of statistical significance in efficacy and confidence.

Although the study included a diverse range of participants in terms of gender, age, and experience, the limited sample size of 20 volunteers restricted the generalizability of the findings to the broader population of interest. A larger sample size may have led to a finding of statistical significance for secondary outcomes. Therefore, limitations in overall enrollment may have been due to this study only occurring at the MSTC cadaver lab and subsequent iterations during the data collection window being canceled. Moreover, our study included a higher number of combat medics in the subgroup of participants. For example, of the 20 participants, two are physician EM staff, one is a physician EM resident, one is a certified PA, three are other medical professionals, and 13 are combat medics. In Lewis et al., all procedures were carried out by fellowship-trained specialist foot and ankle orthopedic surgeons [17], whereas combat medics comprised more than half the sample size of our study. Given this study’s overall small sample size and varying representation of subgroup participants, the generalizability to the population of interest is limited.

As this was a single-center study completed during the MSTC biannual cadaver lab, the investigators had limited available cadavers at that center. To complicate things further, the cadavers were stored in refrigerated trucks, which affected the temperature of the models and the laxity of the skin, limiting the comparison of how DermaClip^®^ would perform on living patients because the adhesive is thermally activated. Also, repeated attempts with sutures on the same cadaver led to needle marks, leading to potential guides for follow-up iterations. Lastly, the MSTC provided a controlled environment without distractions with optimal lighting, which does not emulate austere environments encountered in stressful EDs or while deployed.

Recommendations

The authors have several recommendations for future studies. The goals of laceration management include achieving hemostatic closure and optimizing cosmetic outcomes without increasing the risk of infection and other complications [13].

While this study showed that DermaClip^®^ is faster than conventional sutures in terms of laceration repair, further research is necessary to determine how it compares in terms of cosmetics, infection risk, and complications. We suggest conducting a larger study with a more diverse sample size of providers in different branches of the military and civilians to provide an understanding of how it operates across differing populations. For this study, we used cadaver tissue, which showed a significant difference in time but had limitations due to storage requirements. A follow-up prospective study with live patients is needed to validate study findings and evaluate how DermaClip^®^ will perform in different environments (ED, combat zones, and austere environments). This will also allow for long-term follow-up to assess wound healing, infection rates, scar appearance, and any complications.

The investigators recommend an evaluation of the cost-effectiveness of DermaClip^®^ compared to conventional sutures. The cost-benefit analysis should consider the time saved, the price of each method and ancillary supplies needed, the reduced need for follow-up appointments, and the overall healthcare costs to the patient. This will provide evidence on whether there are actual improvements in cost to facilities.

Lastly, based on the performance of DermaClip^®^ used by medical professionals with minimal laceration repair experience, consideration should be given to integrating DermaClip^®^ into Role I/II/III treatment facilities and carrying it in combat medic aid bags. DermaClip^®^ is lightweight and does not require ancillary equipment to complete laceration repair; it eliminates the need for a topical anesthetic and laceration repair kits, which allows it to be carried by medical personnel at all echelons of care. If DermaClip^®^ gets fielded into these roles of care, research can also be performed on its utility during deployment and efficacy following storage in varying climates.

## Conclusions

This study attempted to demonstrate statistical significance in the time of laceration repair, efficacy, and confidence between DermaClip^®^ and conventional sutures on cadaver models. Following analysis, DermaClip^®^ showed a statistically significant decrease in the time to complete laceration repair that was not replicated when evaluating efficacy and confidence. Although DermaClip^®^ performed better overall, further studies need to be completed on live patients and a cost analysis conducted to determine whether DermaClip^®^ is superior to conventional sutures.
